# Dopamine-Related Genotype Predicts Trajectories of Gait Reserve in Older Adults

**DOI:** 10.1093/geroni/igaa057.2728

**Published:** 2020-12-16

**Authors:** Briana Sprague, Andrea Rosso, Xiaonan Zhu, Caterina Rosano

**Affiliations:** 1 University of Pittsburgh, Pittsburgh, Pennsylvania, United States; 2 School of Public Health, University of Pittsburgh, Pittsburgh, Pennsylvania, United States; 3 University of Pittsburgh Graduate School of Public Health, Pittsburgh, Pennsylvania, United States

## Abstract

The capacity to increase one’s gait speed is critical for maintaining safe community ambulation. There is limited work on the longitudinal changes in this capacity and its predictors. Because lower dopamine is associated with lower task adaptation and motivation, we hypothesized that lower dopamine would predict more decline in rapid gait speed. Catechol-O-methyltransferase (COMT) polymorphism and at least 3 repeated rapid and usual pace gait speed assessments were obtained over 10 years in 1,261 older adults (mean age=75.2, 867 White, 659 women). Linear mixed models computed person-specific rapid and usual pace gait speed trajectories. Regression models adjusted for usual gait trajectory tested whether COMT predicted rapid gait trajectory; covariates included, demographic, psychological, cognitive, and physical factors. Val/Val carriers (lower dopamine) declined more in rapid gait compared to Met/Met carriers (higher dopamine; adjusted b=-.002, SE=.001, p=.042). Modifying dopamine may positively influence the ability to maintain rapid gait over time.

